# Analyzing the impact of induced magnetic flux and Fourier’s and Fick’s theories on the Carreau-Yasuda nanofluid flow

**DOI:** 10.1038/s41598-021-87831-6

**Published:** 2021-04-29

**Authors:** Seemab Bashir, Muhammad Ramzan, Jae Dong Chung, Yu-Ming Chu, Seifedine Kadry

**Affiliations:** 1 Department of Mathematics, Air University, Islamabad, 44000 Pakistan; 2 Department of Computer Science, Bahria University, Islamabad, 44000 Pakistan; 3 Department of Mechanical Engineering, Sejong University, Seoul, 143-747 Korea; 4 Department of Mathematics, Huzhou University, Huzhou, 313000 People’s Republic of China; 5 Hunan Provincial Key Laboratory of Mathematical Modeling and Analysis in Engineering, Changsha University of Science and Technology, Changsha, 410114 People’s Republic of China; 6 Faculty of Applied Computing and Technology, Noroff University College, Kristiansand, 4608 Norway

**Keywords:** Software, Mechanical engineering

## Abstract

The current study analyzes the effects of modified Fourier and Fick's theories on the Carreau-Yasuda nanofluid flow over a stretched surface accompanying activation energy with binary chemical reaction. Mechanism of heat transfer is observed in the occurrence of heat source/sink and Newtonian heating. The induced magnetic field is incorporated to boost the electric conductivity of nanofluid. The formulation of the model consists of nonlinear coupled partial differential equations that are transmuted into coupled ordinary differential equations with high nonlinearity by applying boundary layer approximation. The numerical solution of this coupled system is carried out by implementing the MATLAB solver bvp4c package. Also, to verify the accuracy of the numerical scheme grid-free analysis for the Nusselt number is presented. The influence of different parameters, for example, reciprocal magnetic Prandtl number, stretching ratio parameter, Brownian motion, thermophoresis, and Schmidt number on the physical quantities like velocity, temperature distribution, and concentration distribution are addressed with graphs. The Skin friction coefficient and local Nusselt number for different parameters are estimated through Tables. The analysis shows that the concentration of nanoparticles increases on increasing the chemical reaction with activation energy and also Brownian motion efficiency and thermophoresis parameter increases the nanoparticle concentration. Opposite behavior of velocity profile and the Skin friction coefficient is observed for increasing the stretching ratio parameter. In order to validate the present results, a comparison with previously published results is presented. Also, Factors of thermal and solutal relaxation time effectively contribute to optimizing the process of stretchable surface chilling, which is important in many industrial applications.

## Introduction

Most commonly working liquids and materials, in many engineering disciplines, such as material and chemical processing, possess multifaceted rheological properties, whose viscosity and viscoelasticity can be continuously deformed and reshaped by imposing some forces and external conditions, such as temperature, timescale, stress, and strain. These fluids have the property to be used as heat exchangers and coolant to reduce pumping power. Other than the Newtonian model, these fluids also exhibit a relationship between shear-stress—a strain that makes them completely different. Nowadays, non-Newtonian fluid dynamics are involved in abundant researches due to their practical application. Such liquids have a shear dependent viscosity. The Carreau-Yasuda model is one of the non-Newtonian liquid models which predicts the shear-thinning/thickening behavior and also shows both elevated and low shear levels relationship. Because of this fact, the Carreau-Yasuda model has great industrial and technological applications which include drilling muds, molten polymers, oils, foodstuff, volcanic lava, liquid suspensions, certain paints, polycrystal melts, cosmetic products, and many more. Tanveer et al.^1^] discussed the Carreau-Yasuda nanofluid model in a curved channel with porous space and mixed convection. Carreau-Yasuda nanofluid flow over a nonlinearly stretching sheet in the presence of Joule heating is addressed by Shahid et al.^2^. Khechiba et al.^3^ studied the effect of Carreau-Yasuda rheological parameters in a horizontal porous cavity on subcritical Lapwood convection. Abbasi et al.^4^ explored entropy generation for Carreau-Yasuda nanofluid flow. Hayat et al.^5^ discussed in detail the Carreau-Yasuda fluid flow with Soret and Dufour effects theoretically and numerically. Ahmed et al.^6^ investigated entropy generation to analyze the peristaltic motion of Carreau-Yasuda fluid. Different other features of Carreau-Yasuda nanofluid are elaborated by different researchers^7–9^.

A Magnetic field not only exhibits significant features to regulate the cooling rate but also gives high-quality industrial outputs. Magnetohydrodynamics (MHD) is particularly concerned with the dynamics of electrically conducting fluids. MHD has gained a wide range of attention from many scientists^10–15^ in the field of medicine and science. Ali et al.^16^ highlighted the numerical investigation of the induced magnetic field in mixed convection in the stagnation flow point. They explained when an induced magnetic field is enhanced the transfer rate of heat as well as Skin friction is enhanced. Ramzan et al.^17^ illustrated MHD Carreau flow with nonlinear radiation effects with zero mass surface flux. The significance of MHD and heat sink/source has been observed in many engineering and physical processes. Salahuddin et al.^18^ used a sensor surface in MHD squeezed flow to understand Carreau-Yasuda fluid flow. Hayat et al.^19^ described the radial magnetic field effects on Carreau-Yasuda nanofluid flow using a curved geometry. Tanveer et al.^20^ addressed the influence of the radial magnetic field with velocity and thermal slip effects on the peristaltic flow of the Carreau-Yasuda fluid. They observed the opposite impacts of velocity slip on temperature and velocity distributions. Hayat et al.^21^ explained joule heating and MHD effects on an inclined channel. Malik et al.^22^ elaborated on the Darcy–Forchheimer Carreau-Yasuda nanofluid flow with magnetohydrodynamics. Abdul Hakeem et al.^23^ used a porous medium to explained MHD effects with the heat source and sink term. Hayat et al.^24^ addressed how MHD affects Carreau-Yasuda fluid peristaltic transport in the presence of curved channel and Hall effects. The heat sink and heat source effects considering MHD flow over the vertical stretched sheet was studied by Ibrahim et al.^25^. They examined that the velocity profile and the transfer rate of heat are increased by enhancing the Hartmann number and parameter of mixed convection. Bilal et al.^26^ discussed the exclusive impact of a magnetized viscous fluid in the presence of Hall current over a variably thicked sheet and non-Fourier flux theory. Khan et al.^27^ elaborated the bioconvection MHD Carreau nanofluid flow for the parabolic surface. He used generalized Fourier’s and Fick’s laws to discover the mass and heat flux phenomena.

In fluid mechanics, different researchers^28–32^ put remarkable contributions to understand the mass transfer process by adding Arrhenius activation energy and chemical reaction. It has a large variety of applications in recovery of thermal oil, chemical engineering, in geothermal reservoirs, and cooling of nuclear reactors. Alghamdi^33^ discussed the significance of binary chemical reaction on a nanofluid flow in the presence of Arrhenius Activation Energy with a rotating disk geometry with mixed convection. Dhlamini et al.^34^ studied nanofluid flow with Arrhenius Activation Energy and binary chemical reaction in presence of mixed convection and convective boundary conditions. The meaning of Arrhenius Activation Energy and binary chemical reaction in the presence of heat source/sink was elaborated by Hayat et al.^35^. Analysis of Arrhenius Activation Energy and binary chemical reaction in Couette-Poiseuille nanofluid flow is reported by Ellahi et al.^36^. Hayat et al.^37^ explained binary chemical reaction and Arrhenius activation energy in MHD nanofluid flow with entropy generation minimization. Some recent investigations featuring aspects of activation energy may be found in^38–45^.

In light of the above literature review, this current study is focused to scrutinize the detailed aspects of induced magnetic flux and modified Fourier and Fick's theories on Carreau-Yasuda nanofluid flow with binary chemical reaction and activation energy. Besides, the Newtonian heating condition is incorporated on the boundary to analyze the behavior of the flow. The impact of different physical parameters is also analyzed. Furthermore, the effects of the local Nusselt number and the Skin friction coefficient are addressed. The transport phenomenon is explained by governing equations that are developed including effects of heat generation/absorption in the energy equation. The complicated nonlinear equations are solved using MATLAB solver bvp4c. Graphical results are displayed to discuss the behavior of involved parameters.

## Mathematical formulation

**Figure 1:**
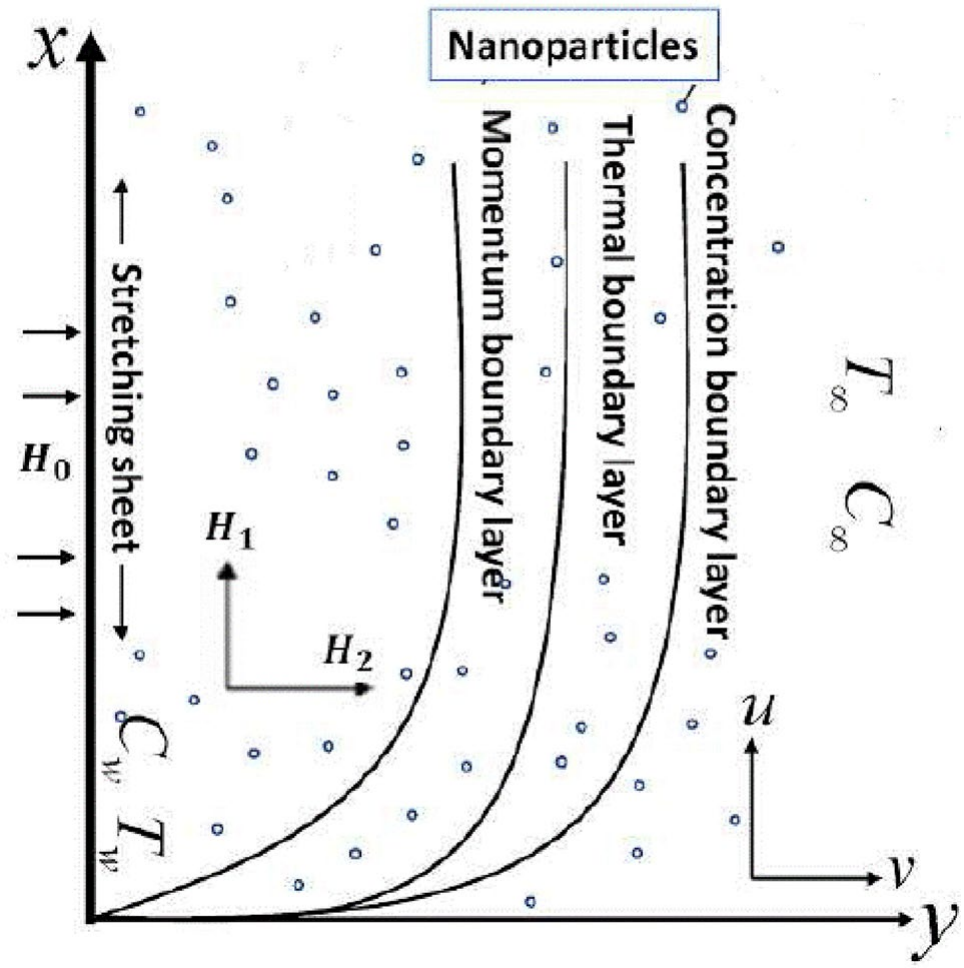
Schematic diagram of the model.

The proposed mathematical model is considered under following assumptions:

1. Here, steady Carreau-Yasuda nanofluid's incompressible flow to a stretching sheet is considered.
2. This current investigation is obtained by assuming the flow with chemical reaction and the Arrhenius activation energy process.
3. To analyze the heat mechanism heat generation/absorption is introduced.
4. The stretched surface is located on $x$- axis and Carreau-Yasuda nano liquid is occupied in the region $y > 0$.
5. Let $u_{e} (x) = ax$ and $u_{w} (x) = cx$. The “$c$” and “$a$” correspond to stretching and free stream velocities respectively.
6. An induced magnetic field $H$ with its parallel component $H_{1}$ and normal component $H_{2}$ is considered here. In Fig. 1 configuration of the flow is plotted.

The Carreau-Yasuda nanofluid model^2,18,19^ is explained by the following equation:

1 $$\tau = \left[ {\mu_{\infty } + (\mu_{0} - \mu_{\infty } )(1 + (\Gamma \dot{\gamma })^{d} )^{{\frac{n - 1}{d}}} } \right]A_{1} ,$$

where $\Gamma ,\,\,\,n$ and $d$ are the parameters used for Carreau-Yasuda nanofluid, $A_{1}$ shows first Rivlin-Ericksen tensor, $\dot{\gamma }$ is represented by $\dot{\gamma } = \sqrt {\frac{1}{2}tr(A_{1} )^{2} } ,$ here $A_{1} = \left[ {grad\,V + (grad\,V)^{T} } \right].$ Assuming $\mu_{\infty } = 0$ transform the above equation into the following form:

2 $$\tau = \left[ {\mu_{0} (1 + (\Gamma \dot{\gamma })^{d} )^{{\frac{n - 1}{d}}} } \right]A_{1} ,$$

Under the above assumptions the model equations are written as^2,18,19,46^:

3 $$u_{x} + v_{y} = 0,$$

4 $$(H_{1} )_{x} + (H_{2} )_{y} = 0,$$

5 $$uu_{x} + vu_{y} - \frac{{\mu_{e} }}{{4\pi \rho_{f} }}\left[ {H_{1} (H_{1} )_{x} + H_{2} (H_{1} )_{y} } \right] = \frac{{\mu_{e} }}{{4\pi \rho_{f} }}H_{e} H_{1}^{\prime } + \frac{{\mu_{f} }}{{\rho_{f} }}u_{yy} - \frac{{(n - 1)(d + 1)\upsilon_{f} }}{d}\Gamma^{d} u_{yy} u_{y}^{d} ,$$

6 $$u(H_{1} )_{x} + v(H_{2} )_{y} - H_{1} u_{x} - H_{2} u_{y} = \mu_{e} (H_{1} )_{yy} ,$$

7 $$\begin{gathered} uT_{x} + vT_{y} + \lambda_{1} (uu_{x} T_{x} + vv_{y} T_{y} + vu_{y} T_{x} + 2uvT_{xy} + u^{2} T_{xx} + v^{2} T_{yy} ) \hfill \\ = \frac{k}{{(\rho c_{p} )_{f} }}T_{yy} + \frac{{Q_{0} }}{{(\rho c_{p} )_{f} }}(T - T_{\infty } ) + \frac{{Q_{0} }}{{(\rho c_{p} )_{f} }}u_{y}^{2} + \frac{{(\rho c_{p} )_{p} }}{{(\rho c_{p} )_{f} }}\left( {D_{B} C_{y} T_{y} + \frac{{D_{T} }}{{T_{\infty } }}T_{y}^{2} } \right), \hfill \\ \end{gathered}$$

8 $$\begin{gathered} uC_{x} + vC_{y} + \lambda_{2} (uu_{x} C_{x} + vv_{y} C_{y} + vu_{y} C_{x} + 2uvC_{xy} + u^{2} C_{xx} + v^{2} C_{yy} ) \hfill \\ = D_{B} C_{yy} + \frac{{D_{T} }}{{T_{\infty } }}T_{y}^{2} - k_{r}^{2} \left( {\frac{T}{{T_{\infty } }}} \right)^{n} e^{{ - \frac{{E_{a} }}{\kappa T}}} \left( {C - C_{\infty } } \right), \hfill \\ \end{gathered}$$

with the following set of conditions on the boundary

9 $$\begin{gathered} u = cx,\,\,v = 0,\,\,(H_{1} )_{y} = H_{2} = 0,\,\, - kT_{y} = h_{f} ( - T + T_{w} ),\,\,D_{B} C_{y} + \frac{{D_{T} }}{{T_{\infty } }}T_{y} \,\,\,\,at\,\,\,y = 0, \hfill \\ u \to ax = u_{e} ,\,\,H_{1} \to H_{0} x = H_{e} x,\,\,C \to C_{\infty } ,\,\,T \to T_{\infty } ,\,\,\,as\,\,y \to \infty . \hfill \\ \end{gathered}$$

Here Eqs. (3) and (4) are the continuity and its corresponding Induced magnetic field equations. Similaraly, Eqs. (5) and (6) represent the momentum and its associated Induced magnetic field equation. However, the heat and concentration equations are numbered as Eqs. (7) and (8), respectively.

Applying transformation

10 $$\begin{gathered} u = cxf^{\prime}(\eta ),\,\,\eta = y\sqrt {\frac{c}{{\upsilon_{f} }}} ,\,\,v = \sqrt {c\upsilon_{f} } f(\eta ), \hfill \\ H_{1} = H_{0} xg^{\prime}(\eta ),\,\,\,H_{2} = H_{0} \sqrt {\frac{c}{{\upsilon_{f} }}} \,\,g(\eta ),\,\,\, \hfill \\ T - T_{\infty } = \theta (\eta )( - T_{\infty } + T_{w} ),\,\,\,C - C_{\infty } = \varphi (\eta )( - C_{\infty } + C_{w} ). \hfill \\ \end{gathered}$$

The model equations are transfigured under the aforementioned transformation into the following form:

11 $$\left( {1 + \frac{(n - 1)(d + 1)}{d}W_{e}^{d} (f^{\prime\prime})^{d} } \right)f^{\prime\prime\prime} + f^{\prime\prime}\,f - f^{{\prime}{2}} + \left( {g^{{\prime}{2}} - gg^{\prime\prime} - 1} \right)\beta = 0,$$

12 $$\lambda g^{\prime\prime\prime} + fg^{\prime\prime} - f^{\prime\prime}g = 0,$$

13 $$\theta^{\prime\prime}\left( {1 - \lambda_{1}^{*} \Pr f^{2} } \right) + \Pr \left( {f\theta^{\prime} + Q\theta + N_{b} \varphi^{\prime}\theta^{\prime} + N_{t} \theta^{{\prime}{2}} - \lambda_{1}^{*} ff^{\prime}\theta^{\prime}} \right) = 0,$$

14 $$\phi^{\prime\prime} + \frac{{N_{t} }}{{N_{b} }}\theta^{\prime\prime} + Sc\left( {f\varphi^{\prime} - \lambda_{2}^{*} \left( {ff^{\prime}\phi^{\prime} + f^{2} \phi^{\prime\prime}} \right)^{\prime \prime } - \sigma \left( {1 + n\delta \theta } \right)e^{{ - \frac{E}{\delta \theta + 1}}} \phi } \right) = 0.$$

With

15 $$\begin{gathered} f^{\prime}(0) = 1,\,\,f(0) = 0,\,\,g(0) = 0,\,\,g^{\prime\prime}(0) = 0,\,\,\theta^{\prime}(0) = - B_{i} [1 - \theta (0)]\,, \hfill \\ N_{b} \phi^{\prime}(0) + N_{t} \theta^{\prime}(0) = 0,\,\,f^{\prime}(\infty ) \to A,\,\,g^{\prime}(\infty ) \to 1,\,\,\theta (\infty ) \to 0,\,\,\phi (\infty ) \to 0. \hfill \\ \end{gathered}$$

The non-dimensional variables are defined as:

16 $$\begin{gathered} A = \frac{a}{c},\,\,\beta = \frac{{\mu_{0} }}{{4\pi \rho_{f} }}\left( {\frac{{H_{o} }}{{c^{2} }}} \right),\,\,\Pr = \frac{{(\mu c_{p} )_{f} }}{{k_{f} }},\,\,\lambda = \frac{{\mu_{e} }}{{\upsilon_{f} }},\,\,\lambda_{1}^{*} = \lambda_{1} c,\,\,\lambda_{2}^{*} = \lambda_{2} c, \hfill \\ Q = \frac{{Q_{0} }}{{c(\rho c_{p} )_{f} }},\,\,W_{e}^{d} = \left( {u_{w} \sqrt {\frac{c}{{\upsilon_{f} }}} \Gamma } \right)^{d} ,\,\,\tau = \frac{{(\rho c_{p} )_{p} }}{{(\rho c_{p} )_{f} }},\,\,N_{t} = \frac{{\tau D_{T} (T_{w} - T_{\infty } )}}{{\upsilon_{f} T_{\infty } }}, \hfill \\ \sigma = \frac{{k_{r}^{2} }}{c},\,\,\delta = \frac{{T_{w} - T_{\infty } }}{{T_{\infty } }},\,\,N_{t} = \frac{{\tau D_{B} (C_{w} - C_{\infty } )}}{{\upsilon_{f} }},\,\,E = \frac{{E_{a} }}{{\kappa T_{\infty } }},\,\,Sc = \frac{{\upsilon_{f} }}{{D_{A} }}. \hfill \\ \end{gathered}$$

## Physical quantities

Physically interesting quantities are to be determined for practical applications in engineering. For instance, the rate of heat transfer $Nu$ and the coefficient of Skin friction $C_{f}$ is mathematically expressed as:

17 $$\,C_{f} = \frac{{2\tau_{w} }}{{\rho_{f} u_{w}^{2} }},\,\,\,\,\,\,\,\,\,\,\,\,\,\,Nu = \frac{{xq_{w} }}{{k_{f} (T_{w} - T_{\infty } )}},$$

in the above equation, the shear stress is $\tau_{w}$ and the heat flux $q_{w}$ is defined as:

18 $$\tau_{w} = \mu_{o} \left[ {1 + \Gamma^{d} \left( {\frac{n - 1}{d}} \right)\left( {\frac{\partial u}{{\partial y}}} \right)^{d} } \right]\left. {\left( {\frac{\partial u}{{\partial y}}} \right)} \right|_{y = 0} ,\,\,\,\,\,\,\,q_{w} = \left. { - \left( {\frac{\partial T}{{\partial y}}} \right)} \right|_{y = 0} .$$

The final expression for the $C_{f}$ and $Nu_{x}$ is written as:

19 $$\,C_{f} {\text{Re}}_{x}^{1/2} = f^{\prime\prime}(0)\left[ {1 + (f^{\prime\prime})^{d} \left( {\frac{n - 1}{d}} \right)W_{e}^{d} } \right],\,\,\,\,\,\,\,Nu_{x} {\text{Re}}_{x}^{1/2} = - \theta^{\prime}(0).$$

in which local Reynold number is ${\text{Re}}_{x}^{1/2} = \frac{{cx^{2} }}{{\upsilon_{f} }}.$

## Numerical scheme

To test the translated coupled non-linear ordinary differential equations, MATLAB program bvp4c is implemented. Using bvp4c, we calculate residuals and carry out the computations for different step sizes $h = 0.01,0.001,....$ As in this process, the absolute convergence requirements were taken $10^{ - 6}$. It is most critical that the necessary finite values of $\eta_{\infty }$ being chosen. For this computational purpose, the asymptotic boundary conditions at $\eta_{\infty }$ for a given case is constrained to $\eta = 5,$ that is enough to better illustrate the solution's asymptotic behavior, of the governed equations. The assumed initial approximation must encounter the BCs without disturbing the solution process. For this first and foremost, fresh variables are added as:

20 $$\begin{gathered} f(\eta ) = y_{1} ,\,\,g(\eta ) = y_{4} ,\,\,\theta (\eta ) = y_{7} ,\,\,\varphi (\eta ) = y_{9} ,\,\,f^{\prime}(\eta ) = y_{2} ,\,\,g^{\prime}(\eta ) = y_{5} ,\,\,\theta^{\prime}(\eta ) = y_{8} , \hfill \\ \varphi^{\prime}(\eta ) = y_{10} ,\,\,f^{\prime\prime}(\eta ) = y_{3} ,\,\,g^{\prime\prime}(\eta ) = y_{6} ,\,\,f^{\prime\prime\prime}(\eta ) = yy_{1} , \hfill \\ g^{\prime\prime\prime}(\eta ) = yy_{2} ,\,\,\theta^{\prime\prime}(\eta ) = yy_{3} ,\,\,\varphi^{\prime\prime}(\eta ) = yy_{4} . \hfill \\ \end{gathered}$$

Using the above expressions in MATLAB bvp4c, a new form of first-order equations is:

21 $$\begin{gathered} yy_{1} = \left[ {\frac{1}{{1 + \frac{(n - 1)(d + 1)}{d}W_{e}^{d} (f^{\prime\prime})^{d} }}} \right]\left[ {y_{2}^{2} - y_{1} y_{3} - \beta \left( {y_{5}^{2} - y_{4} y_{6} - 1} \right)} \right], \hfill \\ \hfill \\ \end{gathered}$$

22 $$yy_{2} = \frac{1}{\lambda }\left( { - y_{1} y_{6} + y_{3} y_{4} } \right),$$

23 $$yy_{3} = \left( {\frac{ - \Pr }{{1 - \lambda_{1}^{*} y_{1}^{2} \Pr }}} \right)[y_{1} y_{8} + Qy_{7} + N_{b} y_{10} y_{8} + N_{t} y_{8}^{2} - \lambda_{1}^{*} y_{1} y_{2} y_{8} ],$$

24 $$yy_{4} = \left( {\frac{ - 1}{{1 - \lambda_{2}^{*} y_{1}^{2} Sc}}} \right)\left[ {\frac{{N_{t} }}{{N_{b} }}yy_{3} + Sc\left( {y_{1} y_{10} - \lambda_{2}^{*} y_{1} y_{2} y_{10} - \sigma \left( {1 + n\delta y_{7} } \right)e^{{ - \frac{E}{{\delta y_{7} + 1}}}} y_{9} } \right)} \right].$$

with the transformed BCs

25 $$\begin{gathered} y_{4} (0) = 0 = y_{6} (0),\,\,y_{2} (0) = 1,\,\,N_{b} y_{10} (0) + N_{t} y_{8} (0) = 0,\,\,y_{8} (0) = - Bi\left[ {1 - y_{7} (0)} \right], \hfill \\ y_{2} (\infty ) = A,\,\,y_{5} (\infty ) = 1,\,\,y_{7} (\infty ) = 0,\,\,y_{9} (\infty ) = 0. \hfill \\ \end{gathered}$$

## Graphical discussion

A graphical interpretation is captured in this section for different embedded physical parameters like $A,\,\,\beta ,\,\,W_{e} ,\,\,n,\,\,\lambda ,\,\,,Q,\,\,Bi,\,\,Sc,\,\,N_{t} ,\,\,N_{b} ,\,\,\lambda_{1}^{*} ,\,\,\lambda_{2}^{*} ,\,\,\delta ,\Pr ,E,$ and $\sigma$^2,18,19,46^ on the physical quantities like velocity, temperature, and concentration. The desired ranges of the these parameters $$\begin{gathered} 2.1 \le A \le 2.4,\,\,0.05 \le \beta \le 0.3,\,\,0.1 \le W_{e} \le 0.4,\,\,1.1 \le n \le 2.0,\,1.3\, \le \lambda \le 1.9,\,\, - 1.2 \le Q \le 1.8, \hfill \\ \,\,1.1 \le Bi \le 2.1,\,\,0.1 \le Sc \le 0.4,\,\,0.1 \le N_{t} \le 0.8,\,\,1.2 \le N_{b} \le 1.8,\,\,1.1\, \le \lambda_{1}^{*} \le 1.8,\,\,1.0 \le \lambda_{2}^{*} \le 1.9, \hfill \\ \,\,0.1 \le \delta \le 1.5,6.2 \le \Pr \le 8.5,0.1 \le E \le 0.9,1.5 \le \sigma \le 1.8. \hfill \\ \end{gathered}$$.

### Velocity and Induced magnetic field profile

**Figure 2:**
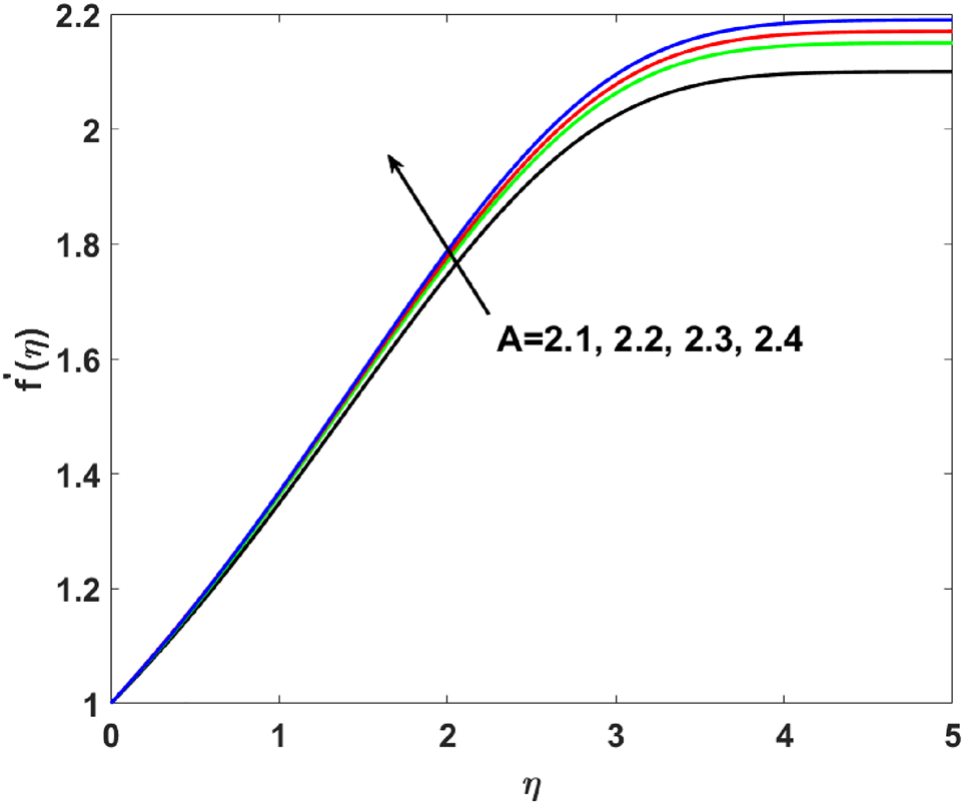
$$f^{\prime}(\eta )$$ versus $$A$$.

**Figure 3:**
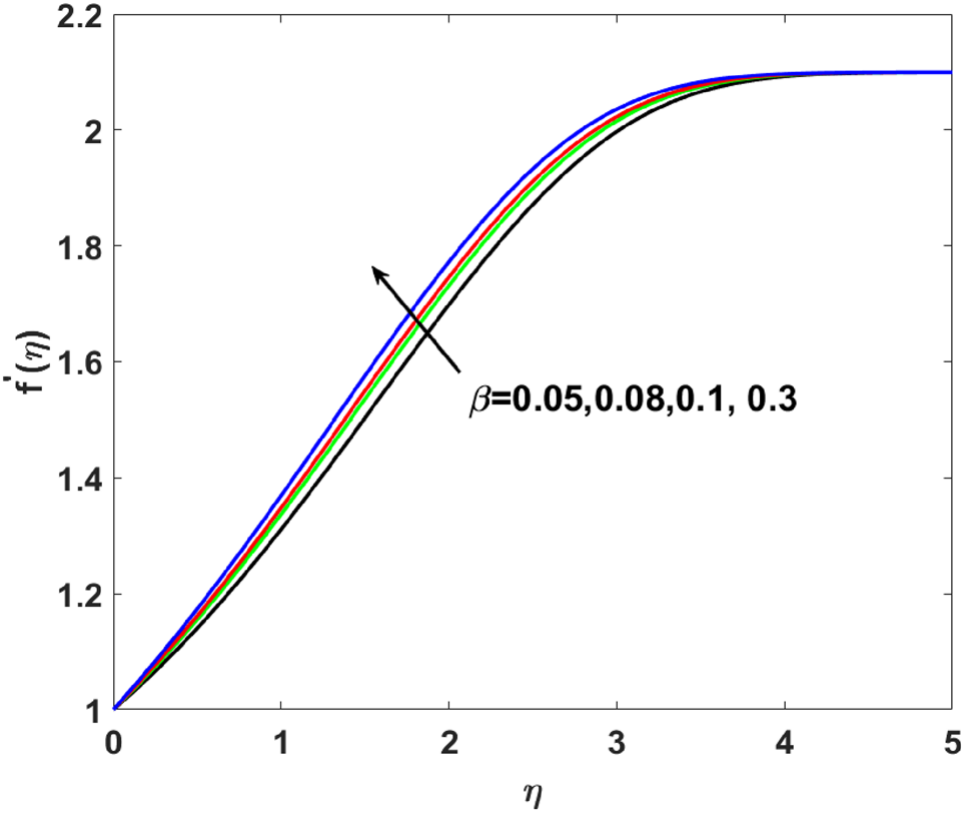
$$f^{\prime}(\eta )$$ versus $$\beta$$.

**Figure 4:**
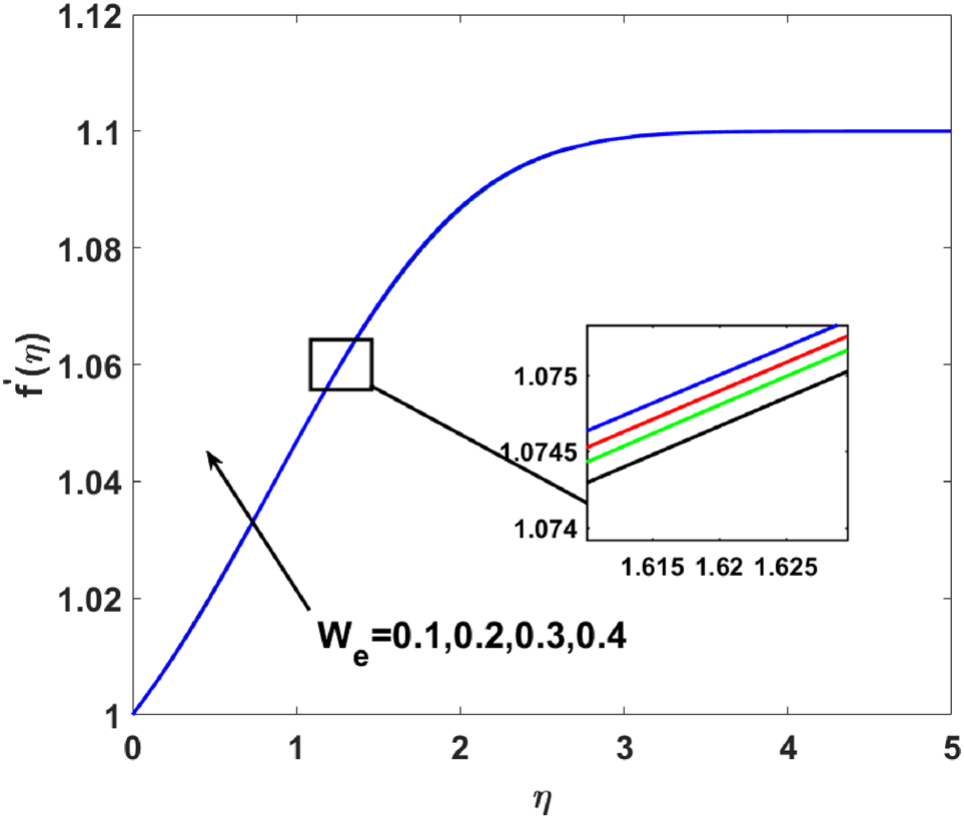
$$f^{\prime}(\eta )$$ versus $$W_{e}$$.

**Figure 5:**
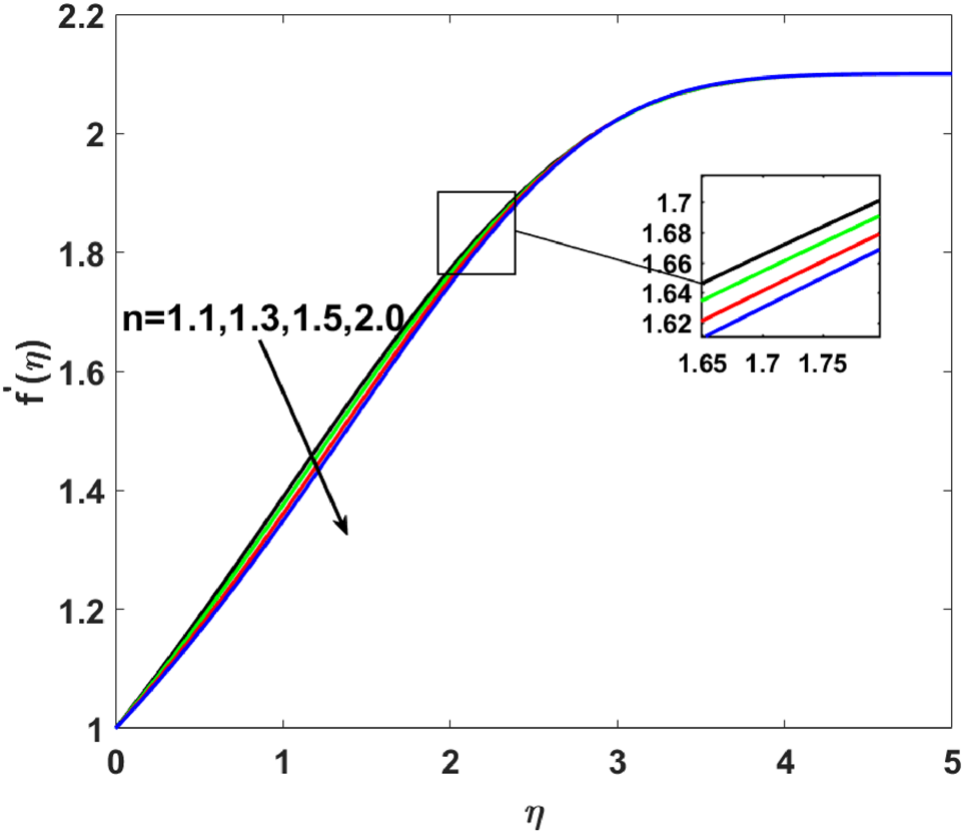
$$f^{\prime}(\eta )$$ versus $$n$$.

**Figure 6:**
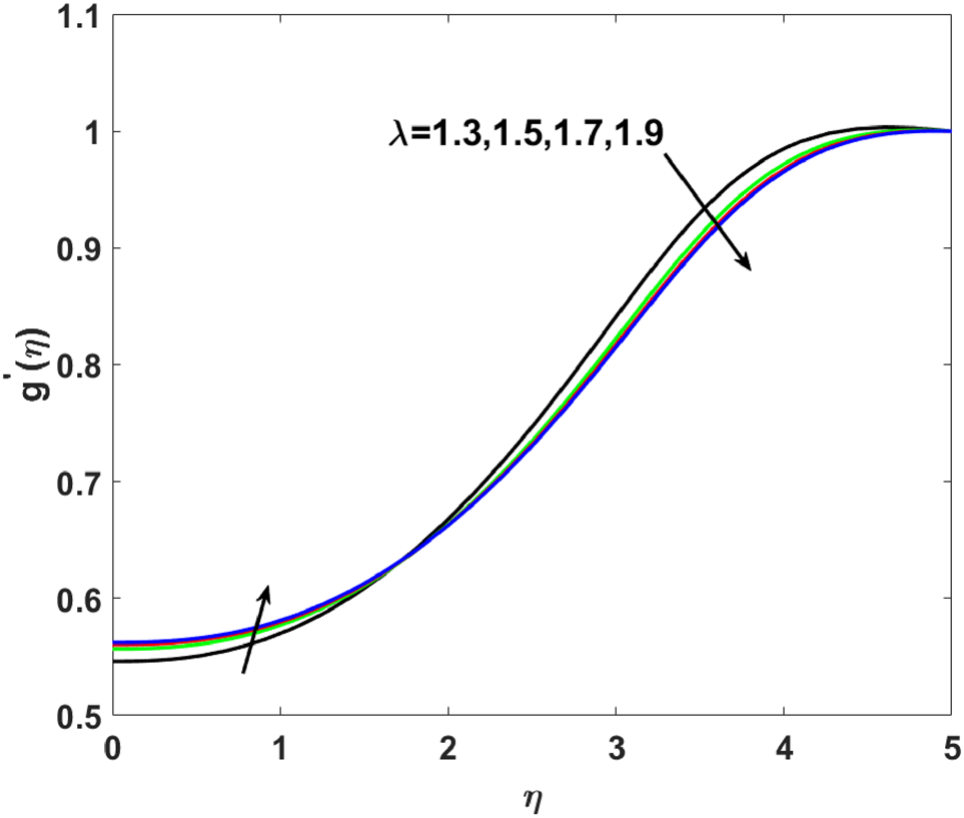
$$g^{\prime}(\eta )$$ versus $$\lambda$$.

The velocity profile is analyzed in Fig. 2 for different values of $$A$$. Increasing trends in the velocity are examined for the mounting values of $$(A = 2.1,2.2,2.3,2.4)$$. It is because of an increase in the value of $$A$$ exhibit more pressure initially, which ultimately increases the velocity and momentum boundary layer.$$f^{\prime}(\eta )$$ is examined for different values of $$\beta$$ $$( = 0.05,0.08,0.1,0.3)$$ in Fig. 3. Enhanced behavior of momentum is noticed on increasing induced magnetic field parameters. Generally, an electric current is developed on an increasing magnetic field which causes the electric force to increase, which, ultimately responsible for the increase in the thickness of both thermal as well as momentum boundary layers. Figure 4 elucidates that the increasing value of $$W_{e}$$$$( = 0.1,0.2,0.3,0.4)$$ increases $$f^{\prime}(\eta )$$. Since Weissenberg is measured as a ratio of the liquid's relaxation time to a given process time. So, its increasing value produces enhancement in relaxation time of the fluid which offers more pressure in flow direction and causes enhancement in the velocity. Figure 5 increases for mounting $$n$$ $$( = 1.1,1.3,1.5,2.0)$$ which causes more resistance in fluid flow. As a result, axial velocity flow declines upon increasing $$n$$. The note $$n = 1$$ represents Newtonian flow behavior. Also, the effects $$\lambda$$ are highlighted on $$g^{\prime}(\eta )$$ in this section. Clearly, Fig. 6 shows that for increasing $$\lambda$$$$( = 1.3,1.5,1.7,1.9)$$ the $$g^{\prime}(\eta )$$ first increases near the surface from $$\eta$$ (0 to 1.9) and then decrease from $$\eta$$ (2 to 5). Induced magnetic boundary layer thickness also increases but $$g^{\prime}(\eta )$$ decreases far from the boundary as the magnetic field is responsible for producing Lorentz force in the flow direction which increases for higher $$\lambda$$.

### Temperature profile

**Figure 7:**
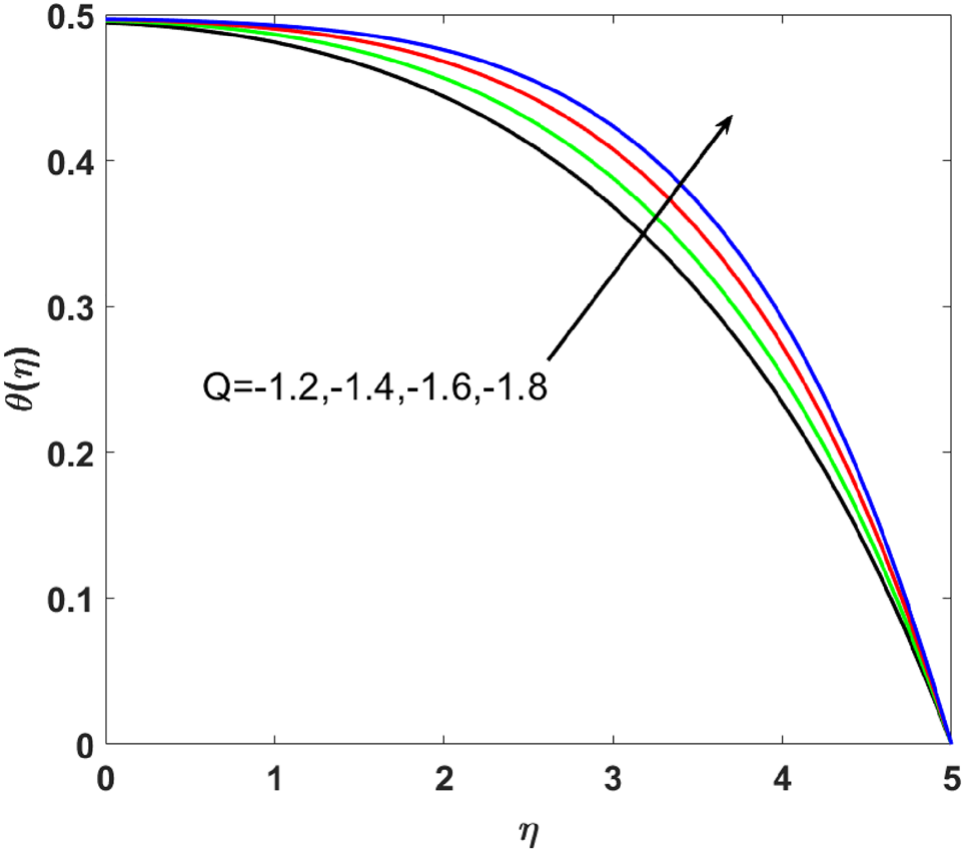
$$\theta (\eta )$$ versus $$Q < 0$$.

**Figure 8:**
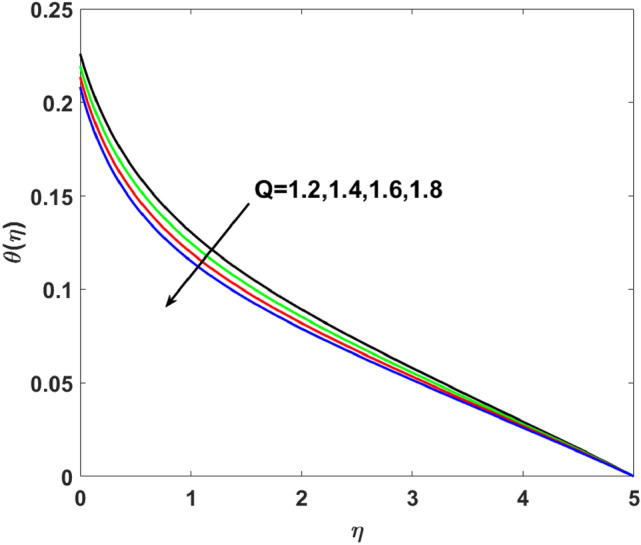
$$\theta (\eta )$$ versus $$Q > 0$$.

**Figure 9:**
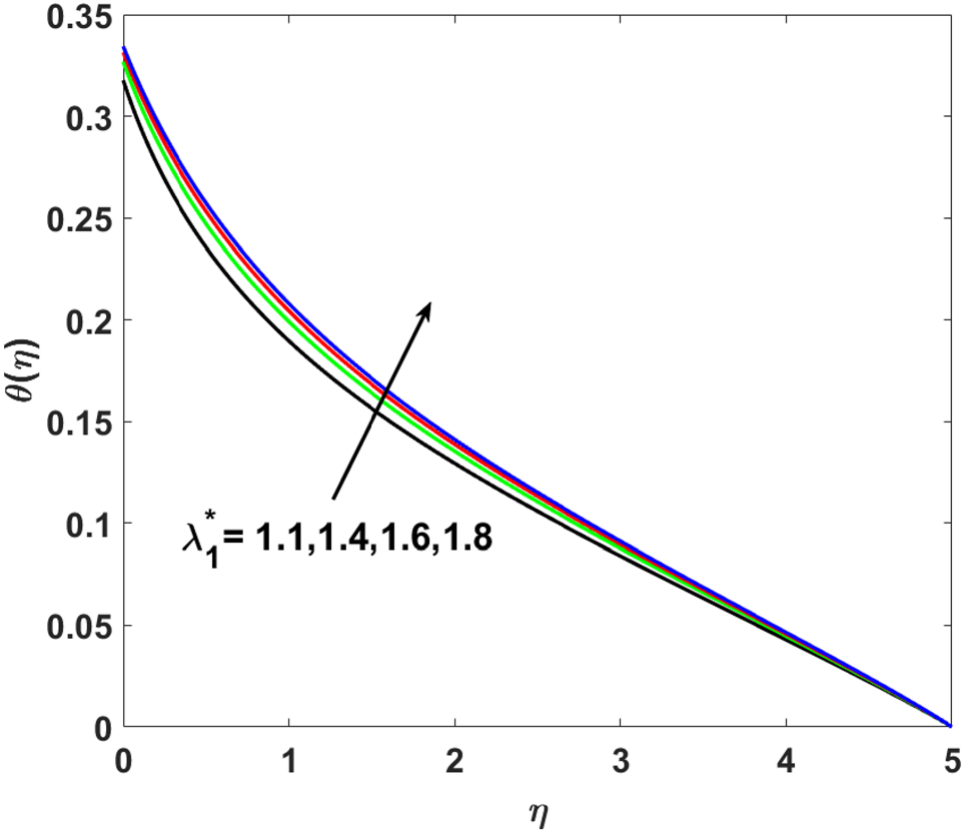
$$\theta (\eta )$$ versus $$\lambda_{1}^{*}$$.

**Figure 10:**
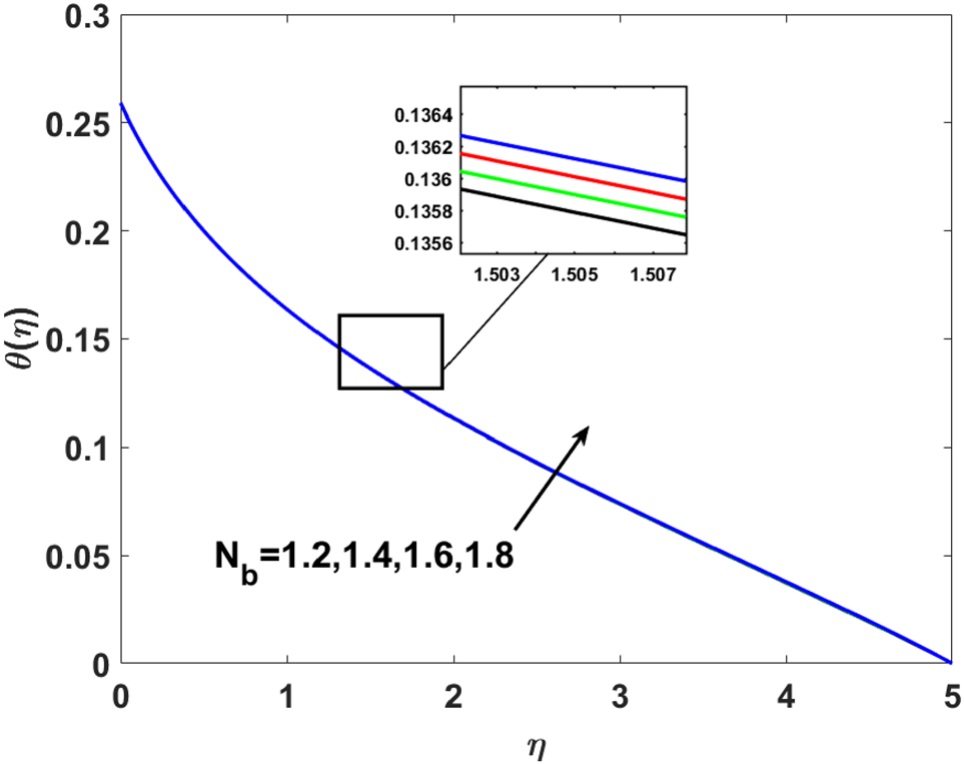
$$\theta (\eta )$$ versus $$N_{b}$$.

**Figure 11:**
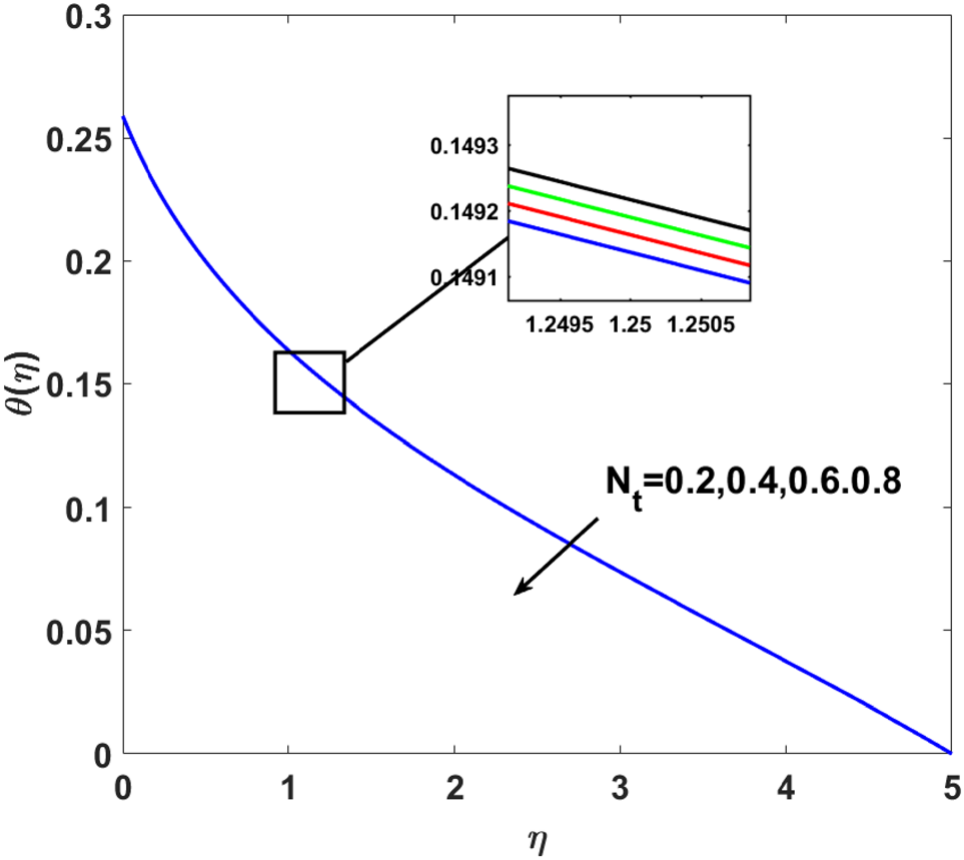
$$\theta (\eta )$$ versus $$N_{t}$$.

**Figure 12:**
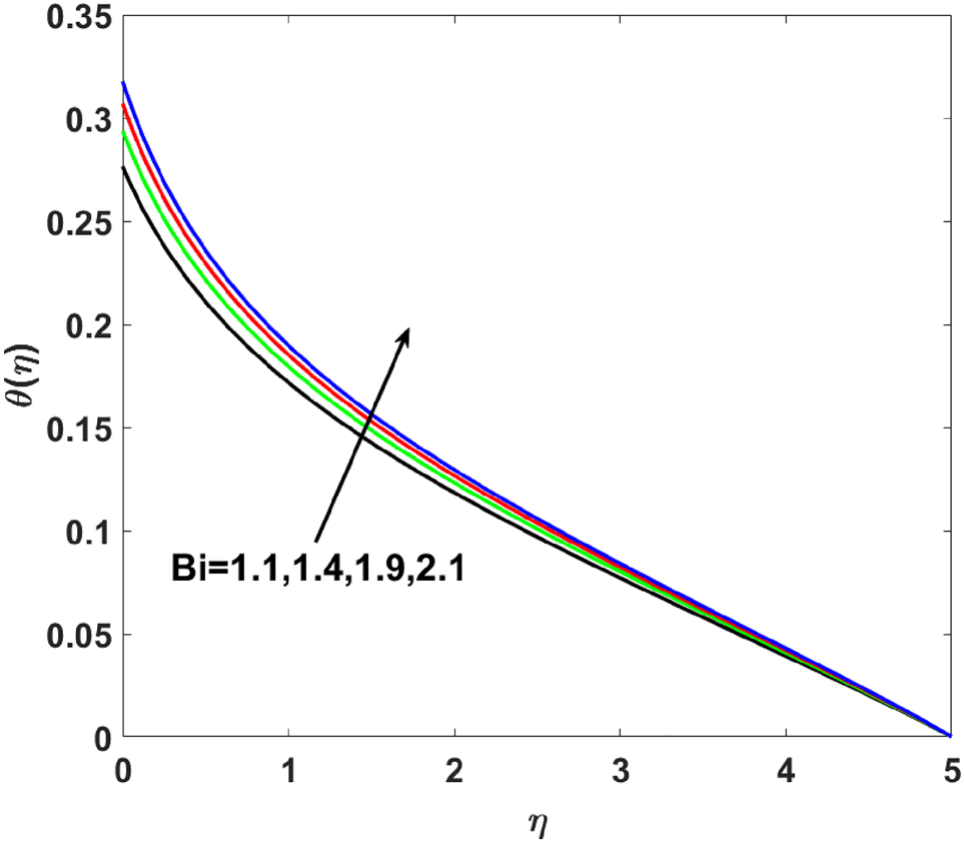
$$\theta (\eta )$$ versus $$Bi$$.

**Figure 13:**
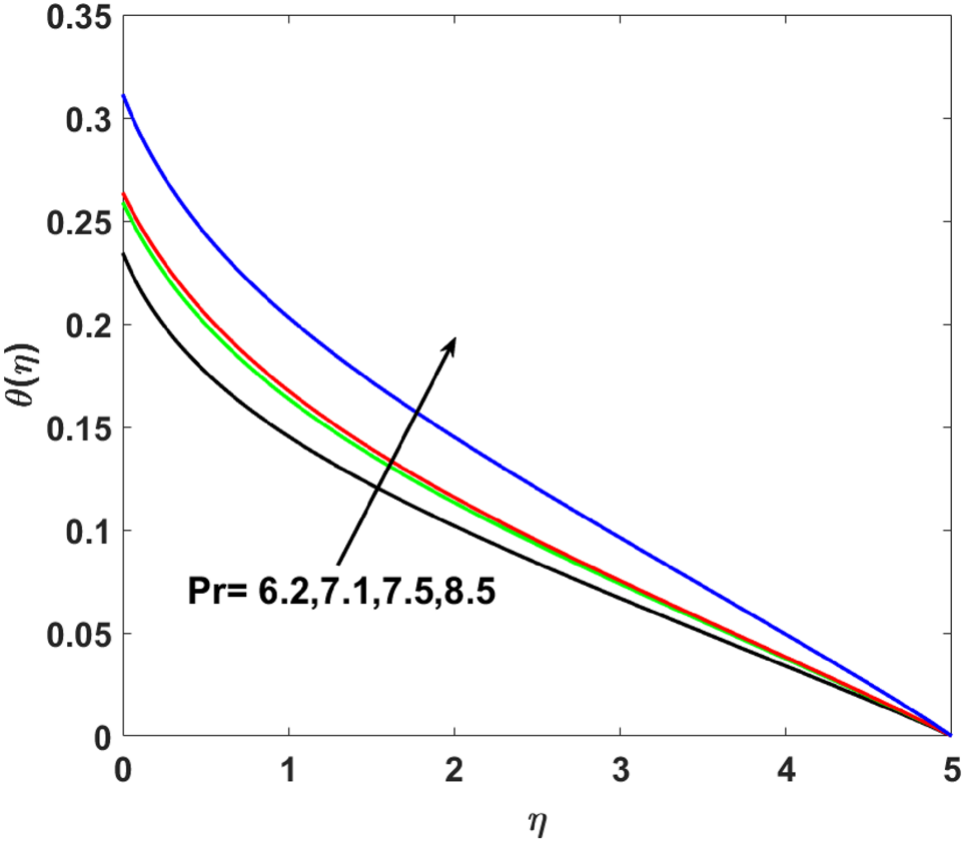
$$\theta (\eta )$$ versus $$\Pr$$.

The effects of $Q,\,\,Bi,\,\,N_{t} ,\,\,N_{b} ,\,\,\lambda_{1}^{*} ,\,\,\Pr$ on $\theta (\eta )$ are explained in this section. The influence of $Q$ on $\theta (\eta )$ for heat sink $(Q < 0),$ and the heat source $(Q > 0)$ is plotted in Figs. 7 and 8. An increase in the temperature of nanofluid is measured for enhancing the value of the heat sink $(Q < 0)$. Whereas, enhancing the value of the heat source reduces the fluid temperature. Sink term puts more energy into layers of a thermal boundary, which leads to a rise in $\theta (\eta )$. Figure 9 shows when $\lambda_{1}^{*}$ increases the thermal distribution $\theta (\eta )$ also increases. Enhancing thermal relaxation time $(\lambda_{1}^{*} = 1.1,\,1.4,\,1.6,\,1.8)$ heat transfer from one particle to another is fast which becomes the reason for the increase in temperature. Figure 10 shows the effects of $N_{b}$. Temperature distribution boosts up due to the random motion of particles for growing values of $(N_{b} = 1.2,1.4,1.6,1.8)$. The response of temperature distribution against the thermophoresis parameter $N_{t}$ is seen in Fig. 11. Physically when $(N_{t} = 0.2,0.4,0.6,0.8)$ rises thermophoretic force increases due to which hot particles move toward cold particles hence temperature rises. Increasing trends of temperature distribution are observed for increasing values of $(Bi = 1.1,1.4,1.9,2.1)$ in Fig. 12. Heat transfer and thermal boundary layer have a direct relation with $Bi$. Therefore, when $Bi$ is increased heat transfer coefficient is increased, and ultimately heat is transferred from the heated source to the cooler surface, which transfers extra heat from the surface to the nanofluid. Hence, $\theta (\eta )$ of nanofluid increases on increasing $Bi$. Similarly, Fig. 13 highlights the effects of $\Pr$. The temperature profile is enhanced for boosting the $\Pr$ $( = 6.2,7.1,7.5,8.5)$ in the flow region. This temperature profile variation is because of large values of $\Pr$ considerably reduces the thermal diffusivity and as a result, the thermal boundary layer thickness reduces which produces an increment in thermal profile.

### Concentration profile

**Figure 14:**
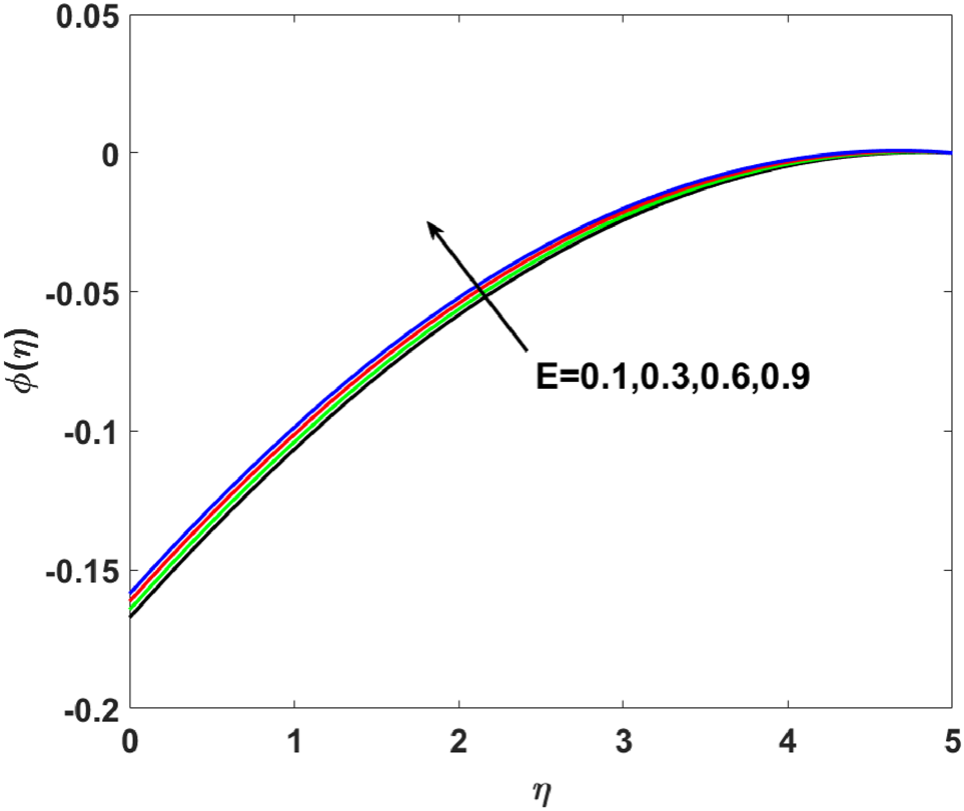
$$\phi (\eta )$$ versus $$E$$.

**Figure 15:**
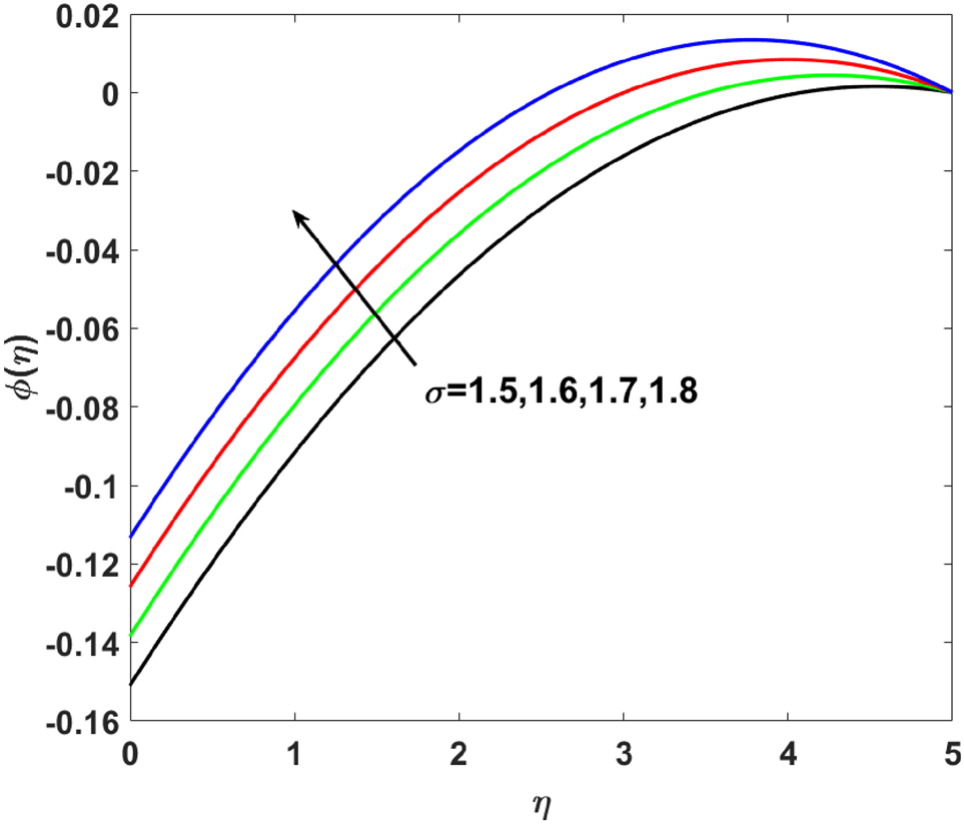
$$\phi (\eta )$$ versus $$\sigma$$.

**Figure 16:**
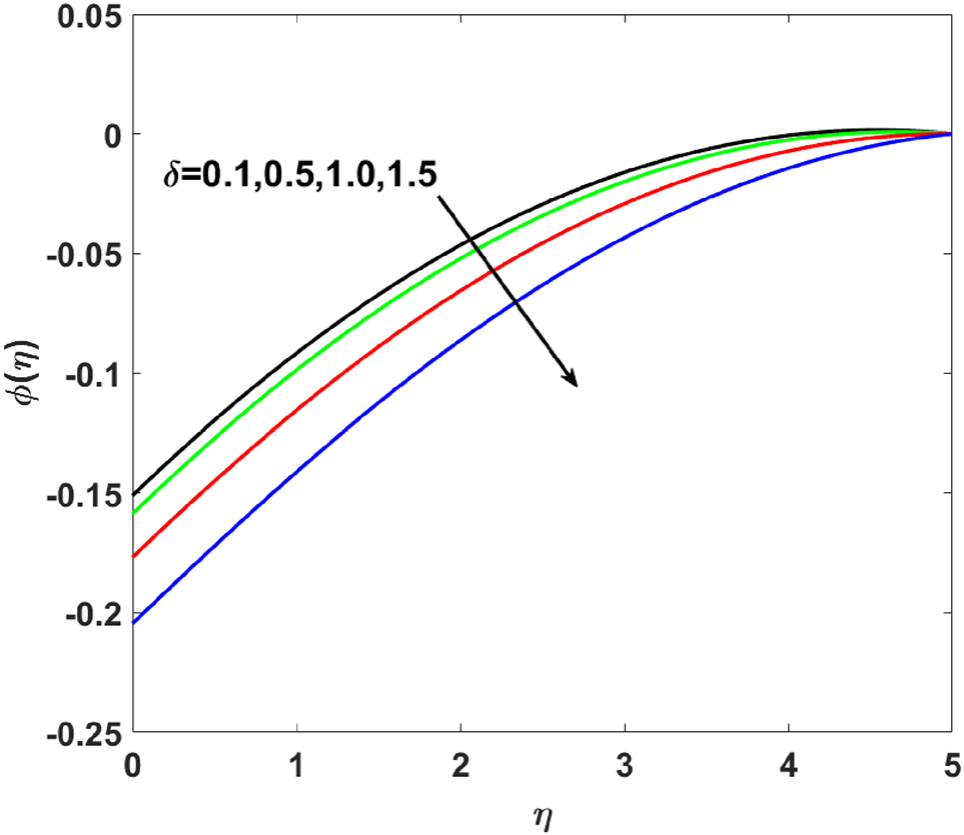
$$\phi (\eta )$$ versus $$\delta$$.

**Figure 17:**
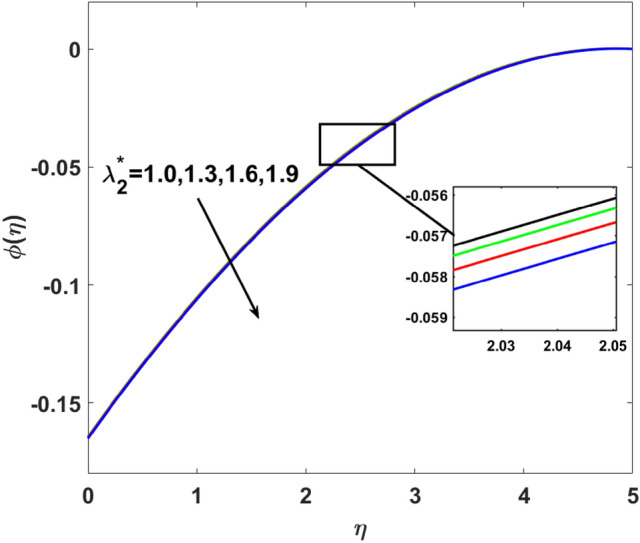
$$\phi (\eta )$$ versus $$\lambda_{2}^{ * }$$.

**Figure 18:**
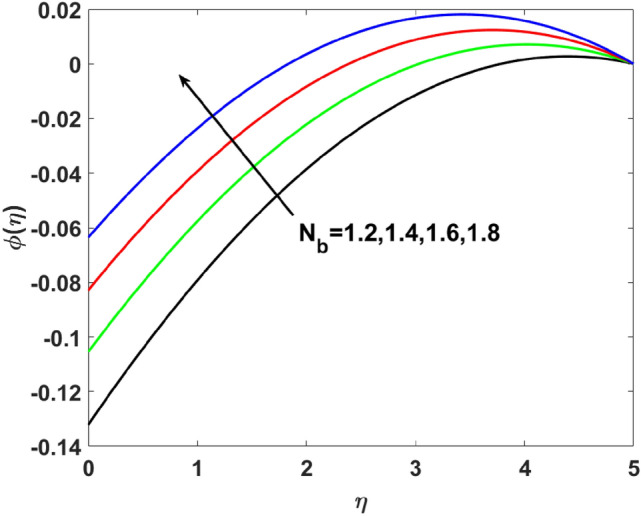
$$\phi (\eta )$$ versus $$N_{b}$$.

**Figure 19:**
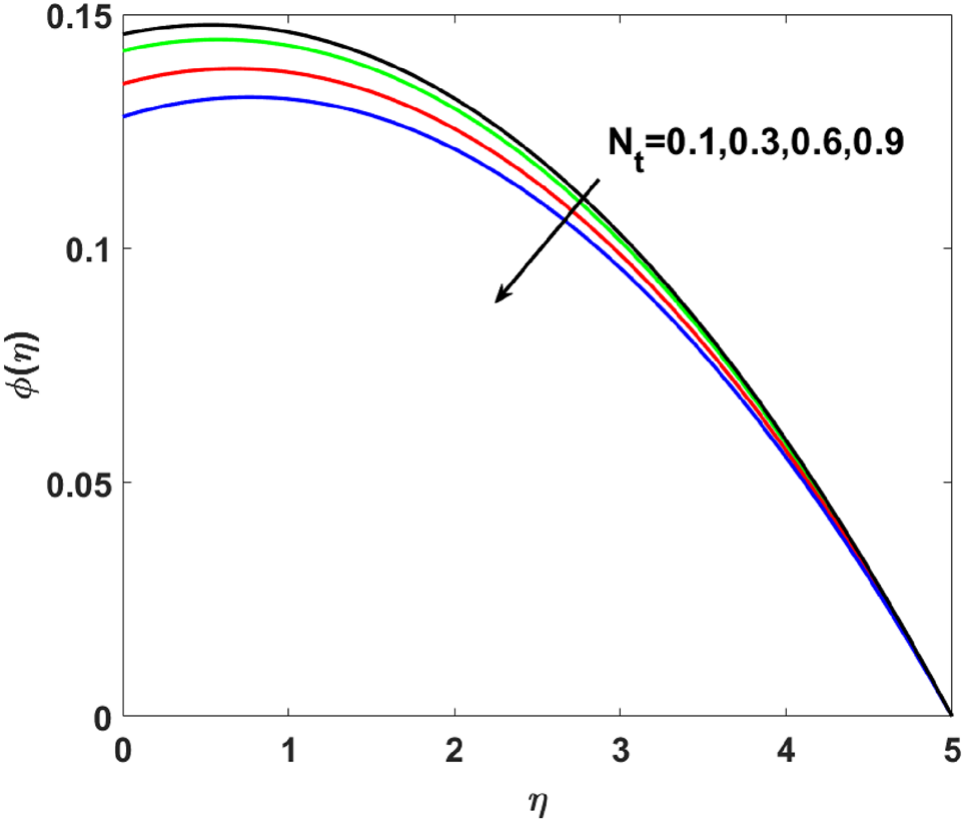
$$\phi (\eta )$$ versus $$N_{t}$$.

**Figure 20:**
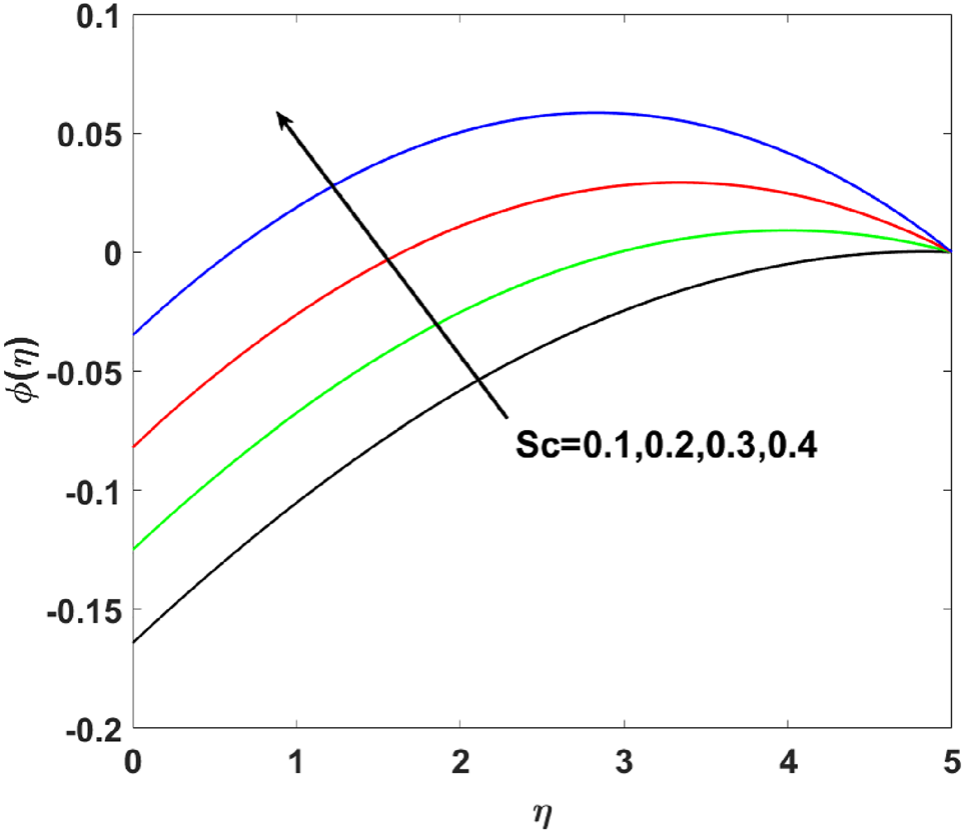
$$\phi (\eta )$$ versus $$Sc$$.

The importance of activation energy $$E$$ is displayed in Fig. 14. The figure shows that the enhancing $$E$$$$( = 0.1,0.3,0.6,0.9)$$, excite thickness of the boundary layer increases the concentration $$\phi (\eta )$$. Figure 15 highlights the impact of $$\sigma$$ on $$\phi (\eta )$$. Observation shows that $$\sigma$$$$( = 1.5,1.6,1.7,1.8)$$ increases both the solutal layer and the concentration field. A higher estimation of $$\sigma$$ is a reason for solutal layer thickness. Therefore, $$\phi (\eta )$$ increases. The effect of $$\delta$$ on $$\phi (\eta )$$ is graphed in Fig. 16. The figure indicates, increasing the value of $$\delta$$ reduces $$\phi (\eta )$$. Figure 17 highlights the effect of the solutal relaxation parameter $$\lambda_{2}^{*}$$ on $$\phi (\eta )$$. A dropped $$\phi (\eta )$$ is observed for large values of $$\lambda_{2}^{*}$$. Both $$N_{b}$$ and $$N_{t}$$ have opposite effects on the $$\phi (\eta )$$ (see Figs. 18, 19). A considerable increase in $$\phi (\eta )$$ is observed when $$N_{b}$$ increases. Moreover, the impact of $$Sc$$ on $$\phi (\eta )$$ is graphed in Fig. 20. The figure indicates that a gradual increase in estimates of the Schmidt number $$Sc$$ results in a thicker boundary layer of concentration. For a large $$Sc$$, concentration diffusivity of fluid is increased which is suppressed for the increasing values of the Schmidt number.

### Skin friction coefficient and heat transfer rate

The impact of different emerging thermophysical parameters on the heat transfer rate as well as on the skin friction coefficient is presented in Tables 1 and 2. Friction factor coefficient increases on escalating Weissenberg number and induced magnetic field parameter whereas reduces for the growing value of stretching ratio parameter $A$. Similarly, the heat transfer rate increases on boosting Thermal relaxation factor $\lambda_{1}^{*}$, $N_{b}$, and the Prandtl number $\Pr$ and declines for increasing heat source/sink parameter $Q$.

**Table 1:** Numerical estimation of $$C_{f} R_{ex}^{{{\raise0.7ex\hbox{$1$} \!\mathord{\left/ {\vphantom {1 2}}\right.\kern-\nulldelimiterspace} \!\lower0.7ex\hbox{$2$}}}}$$ for different parameters.

$\beta$	$n$	$A$	$W_{e}$	$f^{\prime\prime}(0)\left[ {1 + (f^{\prime\prime})^{d} \left( {\frac{n - 1}{d}} \right)W_{e}^{d} } \right]$
0.1	1.1	0.3	1.5	− 0.4172815
0.2				− 0.49195911
0.3				− 0.57265426
0.4				− 0.66085834
	1.2			− 0.53327392
	1.3			− 0.53897413
	1.4			− 0.54478907
		0.4		− 0.35475913
		0.5		− 0.35037484
		0.6		− 0.27708379
			1.6	− 0.42489466
			1.7	− 0.43325409
			1.8	− 0.44241708

**Table 2:** Numerical estimation of $$Nu_{x} R_{ex}^{{{\raise0.7ex\hbox{$1$} \!\mathord{\left/ {\vphantom {1 2}}\right.\kern-\nulldelimiterspace} \!\lower0.7ex\hbox{$2$}}}}$$ for different parameters.

$\lambda_{1}^{ * }$	$N_{t}$	$N_{b}$	$Q$	$\Pr$	$Bi$	$- \theta^{\prime\prime}(0)$
0.2	0.1	0.1	0.3	1.1	0.9	0.13792991
0.3						0.13804022
0.4						0.13818509
0.5						0.13838548
	0.2					0.27877992
	0.3					0.13790234
	0.4					0.13788854
		0.2				0.13795968
		0.3				0.13798945
		0.4				0.13801907
			0.4			0.13784733
			0.5			0.13776472
			0.6			0.13768207
				1.2		0.13794798
				1.3		0.13796686
				1.4		0.13798662
					1.0	0.20425922
					1.1	0.20969592
					1.2	0.2145712

Also, for the accuracy and correctness of the numerical scheme, grid-free analysis for the Nusselt number is presented in Table 3. The current findings of Skin friction coefficient are compared for different values of ${a \mathord{\left/ {\vphantom {a c}} \right. \kern-\nulldelimiterspace} c}$ by Mahapatra and Gupta^47^, Ishak et al.^48^, Nazar et al.^49^, Ali et al.^50^ and Gireesha et al.^51^ in Table 4 by ignoring induced magnetic field effects and Careau Yasuada nanofluid. For each considered value, comparison table displays good agreement, which provides the validity of the correctness and reliability of the latest results.

**Table 3:** Grid free analysis for the Nusselt number.

S. no.	Grid size	$(Nu_{x} )_{ave,\theta }$
1	$10 \times 10$	0.17827252
2	$50 \times 50$	0.17821146
3	$100 \times 100$	0.17826705
4	$300 \times 300$	0.12915111
5	$500 \times 500$	0.12915111
6	$600 \times 600$	0.12915111
7	$1000 \times 1000$	0.12915111

**Table 4:** Comparison of outcomes with Mahapatra and Gupta^47^, Ishak et al.^48^, Nazar et al.^49^, Ali et al.^50^, and Gireesha et al.^51^for co-efficient Skin friction $f^{\prime\prime}(0)$ considering $\beta = n = d = W_{e} = 0$ by varying $A = {a \mathord{\left/ {\vphantom {a c}} \right. \kern-\nulldelimiterspace} c}$.

$A = {a \mathord{\left/ {\vphantom {a c}} \right. \kern-\nulldelimiterspace} c}$	^47^	^48^	^49^	^50^	^51^	Present study
0.1	− 0.9694	− 0.9694	− 0.9694	− 0.9694	− 0.96938	− 0.96933
0.2	− 0.9181	− 0.9181	− 0.9181	− 0.9181	− 0.91810	− 0.91811
0.5	− 0.6673	− 0.6673	− 0.6673	− 0.6673	− 0.66723	− 0.66724
1.0	–	–	–	–	0.90852	0.90853
2.0	2.0175	2.0175	2.0176	2.0175	2.01750	2.01752
3.0	4.7293	4.7294	4.7296	4.7293	4.72928	4.72930
4.0	–	–	–	–	8.00043	8.00046

## Conclusions

In this exploration, we have studied the influence of inclined magnetic flux and modified Fourier and Fick's theories are examined on Carreau-Yasuda nanofluid flow induced by a stretching sheet using the Buongiorno model. Additionally, activation energy with binary chemical reaction is introduced to examine the concentration field. Furthermore, heat source/sink effects are considered along with Newtonian heating on the boundary to analyze the heating mechanism. The final remarks drawn from this study are as follows:

- The growing values of $W_{e}$ decline the thickness of the momentum boundary layer and $f^{\prime}(\eta )$. To boost the rotation parameter, an increase in the axial velocity profile is seen.
- $\theta (\eta )$ boosts on increasing the heat source/sink parameter.
- For broad parameters of $N_{t}$ and $N_{b}$, the temperature of nanofluids is increased.
- A large thermal relaxation factor $\lambda_{1}^{*}$ rises $\theta (\eta )$ and also the thermal boundary layer, whereas larger solutal relaxation factor $\lambda_{2}^{*}$ drops the concentration profile.
- The opposite performance of the concentration boundary layer is observed on increasing $\sigma$ and $\delta$.
- Increasing $E$, boosts the concentration profile.
- The friction factor coefficient increases on escalating Weissenberg $W_{e}$. Whereas, its value reduces for increasing stretching ratio parameter $A$.
- Increasing values of the Thermal relaxation factor and the Prandtl number $\Pr$ boosts the heat transfer rate.

## List of symbols

$$u,\,v$$: Velocity components $${{\text{m}} \mathord{\left/ {\vphantom {{\text{m}} {\text{s}}}} \right. \kern-\nulldelimiterspace} {\text{s}}}$$
$$T$$: Temperature $${\text{K}}$$
$$\mu$$: Dynamic viscosity $${{{\text{m}}^{2} } \mathord{\left/ {\vphantom {{{\text{m}}^{2} } {\text{s}}}} \right. \kern-\nulldelimiterspace} {\text{s}}}$$
$\mu_{f}$: Base fluid dynamic viscosity ${{{\text{m}}^{2} } \mathord{\left/ {\vphantom {{{\text{m}}^{2} } {\text{s}}}} \right. \kern-\nulldelimiterspace} {\text{s}}}$
$H_{e}$: Magnetic field at free stream
$$c_{p}$$: Specific heat $${{\text{J}} \mathord{\left/ {\vphantom {{\text{J}} {{\text{(K}}\,{\text{kg)}}}}} \right. \kern-\nulldelimiterspace} {{\text{(K}}\,{\text{kg)}}}}$$
$$T_{\infty }$$: Ambient temperature $${\text{K}}$$
$D_{B}$: Brownian diffusion coefficient ${{{\text{m}}^{2} } \mathord{\left/ {\vphantom {{{\text{m}}^{2} } {\text{s}}}} \right. \kern-\nulldelimiterspace} {\text{s}}}$
$D_{T}$: Thermophoretic diffusion coefficient ${{{\text{m}}^{2} } \mathord{\left/ {\vphantom {{{\text{m}}^{2} } {\text{s}}}} \right. \kern-\nulldelimiterspace} {\text{s}}}$
$\lambda_{1}$: Relaxation time of heat flux
$q*$: Non-uniform heat source/sink
$\mu_{\infty }$: Infinite shear rate viscosity
$q_{w}$: Wall heat flux ${{\text{W}} \mathord{\left/ {\vphantom {{\text{W}} {{\text{m}}^{2} }}} \right. \kern-\nulldelimiterspace} {{\text{m}}^{2} }}$
$$\beta$$: Magnetic parameter
$$C_{\infty }$$: Ambient concentration $${{{\text{particles}}} \mathord{\left/ {\vphantom {{{\text{particles}}} {\text{L}}}} \right. \kern-\nulldelimiterspace} {\text{L}}}$$
$\lambda_{2}$: RELAXATION time of mass flux ${{{\text{kg}}} \mathord{\left/ {\vphantom {{{\text{kg}}} {{\text{m}}^{2} {\text{s}}}}} \right. \kern-\nulldelimiterspace} {{\text{m}}^{2} {\text{s}}}}$
$C$: Nanoparticle volume fraction ${{{\text{particles}}} \mathord{\left/ {\vphantom {{{\text{particles}}} {\text{L}}}} \right. \kern-\nulldelimiterspace} {\text{L}}}$
$$x,\,y$$: Cartesian coordinates $${\text{m}}$$
$\delta$: Temperature difference variable
$$T_{w}$$: Wall temperature $${\text{K}}$$
$$W_{e}$$: Weissenberg number
$$H_{1} ,\,H_{2}$$: Magnetic components
$\mu_{0}$: Zero shear rate viscosity ${{{\text{m}}^{2} } \mathord{\left/ {\vphantom {{{\text{m}}^{2} } {\text{s}}}} \right. \kern-\nulldelimiterspace} {\text{s}}}$
$$u_{w}$$: Stretching velocity $${{\text{m}} \mathord{\left/ {\vphantom {{\text{m}} {\text{s}}}} \right. \kern-\nulldelimiterspace} {\text{s}}}$$
$u_{e}$: Free stream velocity ${{\text{m}} \mathord{\left/ {\vphantom {{\text{m}} {\text{s}}}} \right. \kern-\nulldelimiterspace} {\text{s}}}$
$$\mu_{e}$$: Rate constant
$$a,\,c,\,C_{o}$$: Dimensional constant
$\rho_{f}$: Base fluid density ${{{\text{kg}}} \mathord{\left/ {\vphantom {{{\text{kg}}} {{\text{m}}^{{3}} }}} \right. \kern-\nulldelimiterspace} {{\text{m}}^{{3}} }}$
$\tau$: Extra stress tensor
$n$: Power law index
$$Sc$$: Schmidt number
$h_{f}$: Heat transmission constant
${\text{Re}}_{x}$: Local Reynolds number
$Q$: Heat generation/absorption
$\lambda$: Reciprocal magnetic Prandtl number
$\theta_{w}$: Temperature ratio parameter
$$\Pr$$: Prandtl number
$A$: Stretching ratio parameter ${\text{m}}$
$\lambda_{1}^{*}$: Thermal relaxation parameter ${\text{s}}$
$\lambda_{2}^{*}$: Solutal relaxation parameter ${\text{s}}$
$$C_{w}$$: Wall concentration $${{{\text{particles}}} \mathord{\left/ {\vphantom {{{\text{particles}}} {\text{L}}}} \right. \kern-\nulldelimiterspace} {\text{L}}}$$
$\sigma$: Non-dimensional activation energy and chemical reaction variable
